# Effects of atelectatic areas on the surrounding lung tissue during mechanical ventilation in an experimental model of acute lung injury induced by lipopolysaccharide

**DOI:** 10.5935/2965-2774.20230190-en

**Published:** 2023

**Authors:** Lídia Maria Carneiro Fonseca, Maycon Moura Reboredo, Leda Marília Fonseca Lucinda, Thaís Fernanda Fazza, Bruno Curty Bergamini, Mateus Pinto Botelho, Gabriele Moura Lopes, Juliana Dias Nascimento Ferreira, Erich Vidal Carvalho, Bruno Valle Pinheiro

**Affiliations:** 1 Pulmonary and Critical Care Division, Hospital Universitário, Universidade Federal de Juiz de Fora - Juiz de Fora (MG), Brazil; 2 Center of Reproductive Biology, Universidade Federal de Juiz de Fora - Juiz de Fora (MG), Brazil

**Keywords:** Acute respiratory distress syndrome, Respiration, artificial, Ventilator-induced lung injury, Pulmonary atelectasis, Lipopolysaccharides, Sepsis, Models, animal

## Abstract

**Objective:**

To assess the effect of atelectasis during mechanical ventilation on the periatelectatic and normal lung regions in a model of atelectasis in rats with acute lung injury induced by lipopolysaccharide.

**Methods:**

Twenty-four rats were randomized into the following four groups, each with 6 animals: the Saline-Control Group, Lipopolysaccharide Control Group, Saline-Atelectasis Group, and Lipopolysaccharide Atelectasis Group. Acute lung injury was induced by intraperitoneal injection of lipopolysaccharide. After 24 hours, atelectasis was induced by bronchial blocking. The animals underwent mechanical ventilation for two hours with protective parameters, and respiratory mechanics were monitored during this period. Thereafter, histologic analyses of two regions of interest, periatelectatic areas and the normally-aerated lung contralateral to the atelectatic areas, were performed.

**Results:**

The lung injury score was significantly higher in the Lipopolysaccharide Control Group (0.41 ± 0.13) than in the Saline Control Group (0.15 ± 0.51), p < 0.05. Periatelectatic regions showed higher lung injury scores than normally-aerated regions in both the Saline-Atelectasis (0.44 ± 0.06 x 0.27 ± 0.74 p < 0.05) and Lipopolysaccharide Atelectasis (0.56 ± 0.09 x 0.35 ± 0.04 p < 0.05) Groups. The lung injury score in the periatelectatic regions was higher in the Lipopolysaccharide Atelectasis Group (0.56 ± 0.09) than in the periatelectatic region of the Saline-Atelectasis Group (0.44 ± 0.06), p < 0.05.

**Conclusion:**

Atelectasis may cause injury to the surrounding tissue after a period of mechanical ventilation with protective parameters. Its effect was more significant in previously injured lungs.

## INTRODUCTION

Patients with acute respiratory distress syndrome (ARDS) depend on mechanical ventilation (MV) to maintain adequate oxygenation and reduce ventilatory work.^(1)^ However, MV can harm the lung by different mechanisms, aggravating tissue inflammation and impairing recovery.^(2,3)^ Strategies that limit tidal volume (V_T_) to 4 - 8mL/kg predicted body weight, plateau pressure (Pplat) < 30cmH_2_O, and driving pressure < 15cmH_2_O are recommended to avoid and minimize this ventilator-induced lung injury (VILI).^(4)^

In ARDS, the lungs present a heterogeneous distribution of aeration, with completely deprived air regions (consolidated and collapsed areas) and normally aerated regions.^(5,6)^ The aerated regions may represent only a small fraction of the lungs in severe forms of ARDS (the baby lung concept).^(7)^ In these cases, VILI may occur even with limited V_T_ due to overdistension of the baby lung, leading to deformation of the extracellular matrix and epithelial and endothelial cells.^(5)^ This overdistension can directly tear the tissue or trigger mechanical transduction signals that initiate an inflammatory cascade.^(8)^

The heterogeneous ARDS lungs also favor the occurrence of VILI due to the excessive and injurious forces generated at the interfaces between opened and closed tissues during MV. Mead proposed this mechanism with a mathematical model that shows that nonatelectatic alveoli are exposed to shear forces from neighboring atelectatic alveoli, which cyclically open and collapse during ventilation. Therefore, atelectasis could act as a concentrator of stress and a trigger for lesions in nearby areas.^(9)^ Based on the mechanisms above, alveolar recruitment strategies might have the potential to reduce VILI, as they increase the amount of alveolar area to receive V_T_, reducing overdistension, and they make the lungs less heterogeneous, reducing areas with an interface between aerated and nonaerated tissues, which are subjected to the highest transpulmonary pressures.

Retamal et al. have already found that there was a greater extent of mechanical trauma and inflammation in the regions surrounding the collapsed areas in an experimental model of atelectasis in rats with initially healthy lungs.^(10)^ Our hypothesis is that the impact of collapsed areas as a stressor on the surrounding areas is more pronounced in previously injured lungs. Therefore, this study aims to analyze injury in the tissue surrounding collapsed lungs during MV in rats with previously injured lungs by intraperitoneal lipopolysaccharide (LPS) injection.

## METHODS

### Animal preparation

Adult male Wistar rats (weighing 307.6 ± 25.9g) were obtained from the Reproduction Biology Center, *Universidade Federal de Juiz de Fora* (UFJF) vivarium (Brazil). Animals received care according to the Principles of Laboratory Animal Care formulated by the National Society for Medical Research. The study was approved by the Ethics Committee in Animal Experiments of the UFJF, Minas Gerais, Brazil.

### Experimental protocol

The animals were initially randomized (in a ratio of 1:1) to receive *Escherichia coli* LPS (LPS serotype 055:B5, purified by phenol extraction, Sigma‒Aldrich, Israel), 10mg/kg dissolved in 0.5mL of 0.9% saline solution (n = 12), or an equivalent amount of saline (n = 12), both intraperitoneally.^(11)^

**Figure 1:**
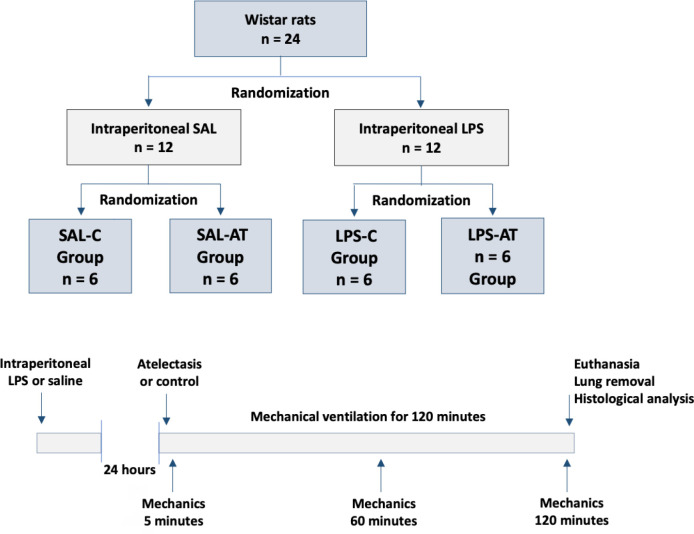
Group allocation and timeline of the study design.

After 24 hours, the rats were anesthetized with an intraperitoneal bolus of ketamine (80mg/kg) and xylazine (8mg/kg). After anesthesia, the rats in both groups (saline and LPS) were randomly assigned to the control or atelectasis groups. Therefore, the following four groups were created: the Saline-Control Group (SAL-C), LPS-Control Group (LPS-C), Saline-Atelectasis Group (SAL-AT), and LPS-atelectasis group (LPS-AT) (Figure 1).

SAL - saline; LPS - lipopolysaccharide; SAL-C - Saline-Control Group; SAL-AT - Saline-Atelectasis Group; LPS-C - Lipopolysaccharide Control Group; LPS-AT - Lipopolysaccharide Atelectasis Group.

A tracheostomy was performed with a 14-gauge catheter, and the right carotid artery was cannulated with an 18-gauge catheter for blood pressure monitoring. In the SAL-AT and LPS-AT Groups, atelectasis was induced by a silicon cylinder blocker (3 mm long and 1.8mm wide). The blocker was attached to an 18-gauge catheter with a metallic guidewire and inserted through the tracheostomy cannula until wedged in the terminal bronchial tree. After wedging the bronchial tree, the block was released by pulling the metallic guidewire through the catheter.^(10)^

The rats were then paralyzed (by 1mg/kg rocuronium 1mg/kg, intravenously) and mechanically ventilated (Inspira ASV, Harvard Apparatus, USA) with the following parameters: V_T_ = 8mL/kg, respiratory rate (RR) = 80 breaths/minute, inspiratory to expiratory ratio = 1:2, fraction of inspired oxygen (FIO_2_) = 0.21, and positive end-expiratory pressure (PEEP) = 5cmH_2_O. After 120 minutes of MV, the animals were euthanized by exsanguination through the carotid line. The trachea was clamped at end-inspiration, and the lungs were removed for further analysis.

### Respiratory system mechanics

Peak airway pressure (Ppeak) was continuously measured with a differential pressure transducer (105124-9, SCIREQ, Montreal, Quebec, Canada) at the distal end of the tracheal cannula. Inspiratory airflow was measured with a heated-controlled pneumotachograph (Hans Rudolph Model 8430B, KS, USA) connected to a pressure transducer (105159-6, SCIREQ, Montreal, Quebec, Canada) and positioned between the tracheal cannula and the Y-piece of the mechanical ventilator. Paw and inspiratory airflow signals were low-pass filtered at 30Hz, digitalized at 1000Hz and recorded with built purpose software (Data Acquisition System, DAS) written in LabVIEW® (National Instruments, Austin, Texas, USA). Tidal volume was calculated by numerical integration of inspiratory airflow.^(12)^

### Lung histology

Lungs were removed in blocks, and atelectases were identified by macroscopic examination. Two regions of interest were defined: the periatelectatic region (defined as 3 mm of tissue surrounding the atelectasis) and the lung contralateral to the atelectasis (lower right lobe or lower portion of the left lung). The regions of interest were isolated, fixed in 10% buffered formaldehyde, and processed for paraffin embedding. To normalize the regions of interest, six sequential 4µm thick slices were cut until the atelectatic and periatelectatic regions distal to the airway obstructed by the silicon blocker or until the normally-aerated pulmonary parenchyma distal to the opened airway in the atelectasis and control groups, respectively. The slices were then stained with hematoxylin-eosin, and morphological examinations were performed by an investigator who was blinded to the study groups with a conventional light microscope (Zeiss, Hallbergmoos, Germany).

Lung injury was quantified using a modified weighted scoring system, as described elsewhere. Briefly, 10 random fields at a magnification of 400X were independently scored in both periatelectatic and normally-aerated areas. Values of zero, one or two were used to represent the severity based on the following findings: neutrophils in the alveolar space, neutrophils in the interstitial space, hyaline membranes, proteinaceous debris filling the airspaces, and alveolar septal thickening. To generate a lung injury score, the sum of the five variables was weighted according to the relevance ascribed to each one. The resulting score was a continuous value between zero (normal) and one (the most severe injury). Additionally, the extent of each lung injury score component was calculated based on the sum of the values (zero, one, or two) of each of the ten analyzed fields.^(13)^

### Statistical analysis

The normality of the data was analyzed by the Kolmogorov‒Smirnov test. Parametric data are expressed as the mean ± standard deviation, and nonparametric data are expressed as the median (interquartile range). One-way ANOVA followed by Tukey’s test was used to compare parametric data. For nonparametric data, the Kruskal‒Wallis test was used followed by the Mann‒Whitney U test. Two-way analysis of variance for repeated measures was applied to evaluate the effects of time and group differences on respiratory variables. In *post hoc* analysis, to separate differences between means, we used the Tukey pairwise multiple-comparison test when a significant F ratio was obtained for a factor or for an interaction of factors. Adjustments for repeated comparisons were performed according to the Bonferroni correction. A p value < 0.05 was considered significant. All statistical analyses were performed using Statistical Package for the Social Sciences (SPSS) 18.0 for Windows (SPSS Inc., Illinois, USA).

## RESULTS

Thirty-six rats were divided into four groups. Seven rats died during the MV period: three from the LPS-C Group, two from the LPS-AT Group, one from the SAL-C Group and one from the SAL-AT Group. Three rats from the LPS-AT Group and two rats from the SAL-AT Group were also excluded because, at the end of the experiments, macroscopic analysis of the lungs showed that atelectasis was not successfully induced. Among the rats that were included in the study, histological analysis showed that the introduction of emboli through the airways caused the development of a small area of atelectasis, present in the right lower lobe in ten rats (83%) and in the lower portion of the left lung in two rats (17%). These positions were confirmed during the histological analysis by the visualization of the blocker in the airway proximal to the atelectatic area.

### Respiratory mechanics

Respiratory system mechanics are shown in table 1. Elastance of the respiratory system (Ers) increased in the four groups throughout the experiment without a significant relationship between time and group. The Ers was higher in the LPS-AT Group than in the SAL-C and LPS-C Groups at 60 and 120 minutes. No significant differences in the resistance of the respiratory system (Rrs), V_T_/kg or RR were observed among the four groups (Table 1). No significant differences in the volume of fluid received during the experiments were observed among the four groups (Table 1).

**Table 1:** Respiratory mechanics and volume of fluid infused during the two-hour period of mechanical ventilation

Respiratory measurements by group	Time after start of protocol			p value		
	Baseline	60 minutes	120 minutes	Group	Time	Interaction
V_T_ /kg (mL/kg)						
SAL-C	7.9 ± 0.2	7.7 ± 0.3	7.7 ± 0.4	NS	NS	NS
SAL-AT	7.6 ± 0.4	7.6 ± 0.5	7.8 ± 0.7			
LPS-C	7.6 ± 0.3	7.7 ± 0.5	7.7 ± 0.2			
LPS-AT	7.8 ± 0.3	7.5 ± 0.4	7.5 ± 1.3			
RR (bpm)						
SAL-C	76.3 ± 0.8	76.6 ± 0.7	76.6 ± 0.7	NS	NS	NS
SAL-AT	76.0 ± 1.1	76.1 ± 1.6	76.3 ± 0.9			
LPS-C	75.8 ± 0.9	76.1 ± 0.8	76.0 ± 0.8			
LPS-AT	7.4 ± 0.4	76.4 ± 1.0	76.1 ± 0.8			
Ppeak (cmH_2_O)						
SAL-C	10.8 ± 1.0	11.0 ± 0.8	11.4 ± 0.8	NS	< 0.01	NS
SAL-AT	11.4 ± 0.7	11.7 ± 1.3	12.1 ± 1.3†			
LPS-C	10.3 ± 0.4	10.7 ± 1.0	10.5 ± 0.7			
LPS-AT	11.9 ± 1.3†	12.7 ± 0.7^*^†	13.2 ± 0.5^*^†			
PEEP (cmH_2_O)						
SAL-C	3.8 ± 0.4	3.6 ± 0.4	3.6 ± 0.3	NS	NS	NS
SAL-AT	3.6 ± 0.3	3.6 ± 0.1	3.5 ± 0.1			
LPS-C	3.9 ± 0.9	3.9 ± 0.4	3.8 ± 0.6			
LPS-AT	3.9 ± 0.8	3.9 ± 0.9	3.9 ± 0.8			
Ers (cm H_2_O/l)						
SAL-C	2.4 ± 0.4	2.6 ± 0.4 ‡	2.8 ± 0.4 §‡	0.017	< 0.01	NS
SAL-AT	2.9 ± 0.5	3.1 ± 0.6 ‡	3.4 ± 0.7 §‡			
LPS-C	2.3 ± 0.2	2.5 ± 0.3 ‡	2.6 ± 0.3 §‡			
LPS-AT	3.1 ± 0.6	3.4 ± 0.5^*^† ‡	3.6 ± 0.5^*^† §‡			
Rrs (cmH_2_O/l/s)						
SAL-C	0.10 ± 0.01	0.09 ± 0.00	0.10 ± 0.03	NS	NS	NS
SAL-AT	0.11 ± 0.02	0.10 ± 0.02	0.10 ± 0.02			
LPS-C	0.12 ± 0.05	0.15 ± 0.10	0.11 ± 0.39			
LPS-AT	0.12 ± 0.03	0.15 ± 0.07	0.18 ± 0.13			
Volume of fluids infused (mL)						
SAL-C			3.71 ± 0.87	NS		
SAL-AT			3.22 ± 0.58			
LPS-C			3.03 ± 0.41			
LPS-AT			3.62 ± 0.58			

SAL-C - Saline-Control Group; LPS-C - Lipopolysaccharide Control Group; SAL-AT - Saline-Atelectasis Group; LPS-AT - Lipopolysaccharide Atelectasis Group.

* p < 0.05 compared to Saline-Control Group; † p < 0.05 compared to normalized lung region within the same group; ‡ p < 0.05 compared to the same region of the Saline-Atelectasis Group. Statistical analysis was performed using one-way ANOVA followed by Tukey’s test or the Kruskal-Wallis test followed by the Mann-Whitney U test for normally and nonnormally distributed data, respectively. Adjustments for repeated measures were performed according to the Bonferroni correction. Values are expressed as the mean ± standard deviation or median (interquartile range) for normally and nonnormally distributed data, respectively.

### Histological analysis

**Figure 2:**
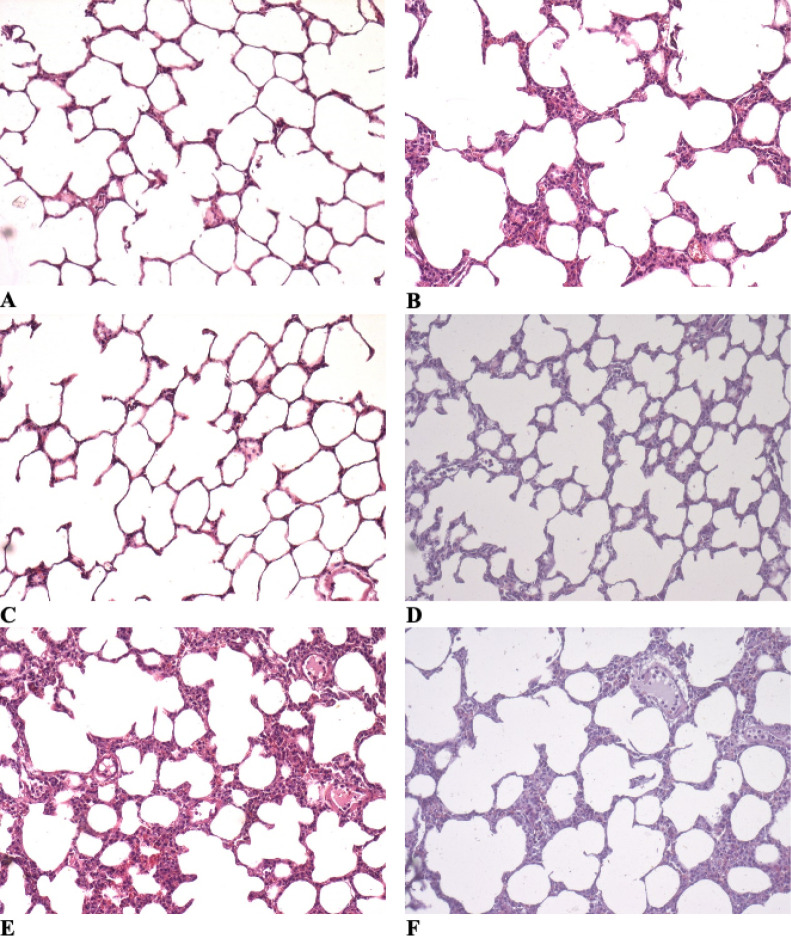
Photomicrographs of lung parenchyma stained with hematoxylin-eosin x 400.

Atelectasis was evident in the lungs of the animals from the SAL-AT and LPS-AT Groups, confirming the ex vivo macroscopic analysis. Rats from the LPS-C Group showed a higher acute lung injury (ALI) score than those from the SAL-C Group. The analysis of each component of the score demonstrated that rats from the LPS-C Group had greater alveolar and interstitial neutrophil infiltration, as well as a greater amount of alveolar proteinaceous debris (Table 2, Figure 2).

**Table 2:** Acute lung injury score and its components

	Groups					
	SAL-C	LPS-C	SAL-AT		LPS-AT	
			Normally-aerated	Periatelectatic	Normally-aerated	Periatelectatic
Overall score	0.15 ± 0.51	0.41 ± 0.13^*^	0.27 ± 0.74	0.44 ± 0.06†	0.35 ± 0.04	0.56 ± 0.09†‡
Alveolar neutrophils	0 (0.75)	3.00 (8.75)	2.50 (4.00)	7.00 (5,75)†	3.00 (3.25)	12.50 (7.00)†
Interstitial neutrophils	9.33 ± 2.33	19.50 (1.75)^*^	14.00 ± 2.44	17.83 ± 1.16†	18.83 ± 1.47	19.16 ± 1.16
Proteinaceous debris	2.00 (1.50)	6.66 ± 3.55	1.50 (1.50)	6.00 (8.75)†	3.00 (1.75)	7.00 (4.25)†
Hyaline membrane	00	00	00	00	00	00
Septal thickening	0.00 (0.25)	1.83 ± 3.12	0.00 (0.25)	0.00 (1.00)	0.50 (1.25)	2.50 ± (6.25)

V_T_ - tidal volume; SAL-C - Saline-Control Group; NS - not significant; LPS-C - Lipopolysaccharide Control Group; SAL-AT - Saline-Atelectasis Group; LPS-AT - Lipopolysaccharide Atelectasis Group; RR - respiratory rate; Ppeak - peak airway pressure; PEEP - positive end-expiratory pressure; Ers - elastance of the respiratory system; Rrs - resistance of the respiratory system.

* p < 0.05 compared to Saline-Control Group; † p < 0.05 compared to Lipopolysaccharide Control Group; ‡ p < 0.05 compared to baseline of the same group; § p < 0.05 compared to 60 minutes of the same group. Data are expressed as the mean ± standard deviation.

(A) Saline-Control Group; (B) Lipopolysaccharide Control Group; (C) Saline-Atelectasis Group, normally-aerated lung region; (D) Saline-Atelectasis Group, periatelectatic lung region; (E) Lipopolysaccharide Atelectasis Group, normally-aerated lung region; (F) lipopolysaccharide atelectasis group, periatelectatic lung region. (A and C) normal lung; (B, D, E and F) alveolar wall thickening, neutrophils in the interstitium and in the airspace, proteinaceous debris in the airspace.

In both groups of rats with atelectasis (SAL-AT and LPS-AT), peri-atelectasis regions showed a higher ALI score than normal aerated regions from the contralateral lungs. Peri-atelectasis regions had greater alveolar and interstitial neutrophil infiltration and a greater amount of alveolar proteinaceous debris. Rats from the LPS-AT Group showed a higher ALI in the peri-atelectasis regions than rats from the SAL-AT Group (Table 2, Figure 2).

## DISCUSSION

In this study, we applied a nonlobar atelectasis model developed by Retamal et al. to investigate lung injury at the interface between opened and collapsed lung regions in rats under MV.^(10)^ To apply our findings to clinical practice, we evaluated the effect of atelectasis in previously injured lungs by intraperitoneal injection of LPS, and we ventilated the animals according to ventilatory parameters commonly used in clinical practice (V_T_ of 8mL/kg and PEEP of 5cmH_2_O). Lung injury was more significant in the regions surrounding atelectatic areas than in normally aerated regions, confirming our hypothesis that collapsed areas may amplify the injury induced by MV.

In ARDS, lung volumes are heterogeneously distributed, and there are atelectatic areas near both normally aerated and even overdistended areas. These inhomogeneities increase local stress and strain and may promote VILI.^(5,9)^ Theoretical foundations suggest that at the interface between a fully opened and a fully closed area, transpulmonary pressure rises to values much higher than those reached in homogeneous lungs. This repeated exposure to high transpulmonary pressure, due to the cyclic closure and reopening of alveoli during the respiratory cycle, contributes to VILI and is called atelectrauma.^(9,14)^

To analyze the impact of atelectasis on VILI occurrence, we used the experimental model described by Retamal et al. In this model, an isolated peripheral atelectatic area is obtained by bronchial blocking with a silicon cylinder blocker, creating an interface between collapsed and opened alveolar areas. They observed histological evidence of lung injury and inflammation in the peri-atelectasis regions, suggesting that atelectasis acts as a stress concentrator.^(10)^ However, in their study, the rats had previously healthy lungs before the experiments and were treated with 20mL/kg V_T_, conditions that are unlikely to be found in clinical practice.

To better replicate the clinical scenario of VILI during MV due to ALI, we created an atelectasis model in rats with ALI induced by intraperitoneal LPS injection. This model of ALI is well established and causes mild and transitory inflammation in lung tissue. In contrast to other ALI models, particularly the one obtained by repeated lung lavages, our model does not lead to extensive alveolar collapse or heterogeneity in the distribution of lung volumes.^(15)^ This feature avoids the occurrence of tidal recruitment caused by extensive collapsed areas that could lead to overdistension of opened alveoli. Thus, it is possible to isolate the effect of the atelectasis caused by this model by comparing opened and collapsed lungs. Our ALI model reproduced the features described in the literature with the presence of inflammation and the absence of impaired respiratory mechanics. The animals in the LPS-C Group showed more severe lung injury, as evidenced by the presence of more interstitial and alveolar neutrophils and more proteinaceous debris compared with the SAL-C Group. There was no difference in Esr at baseline or during the two-hour period of MV among these groups.

In our study, atelectasis acted as a stressor and caused increased lung injury during MV despite the low V_T_ of 8mL/kg that was applied. Periatelectatic areas in both the saline and LPS groups were more injured, as shown by a higher lung injury score and by more interstitial, alveolar and proteinaceous debris compared to the contralateral normalized areas. The effect of atelectasis on VILI was more severe in the LPS groups, which means that the lungs that had been previously primed by inflammation were more easily injured. This finding might be explained by the two-hit hypothesis whereby two insults act synergically to cause injury.^(16,17)^ In this study, the inflammation caused by intraperitoneal LPS might have prepared the innate immune system for a more rapid and significant response to the increase in stress caused by atelectasis. In fact, a combination of these models might be a better representation of the complex and multifactorial pathophysiology of ARDS whereby an initial injury is likely to increase the need for MV.^(18)^

There are some limitations in our study that must be considered. First, the atelectasis model does not reflect the magnitude and the site of lung collapse that occurs in ARDS. Second, the model of ALI induced by intraperitoneal LPS causes mild and transitory lung inflammation that does not reproduce the pathologic aspects of ARDS. Third, although we excluded samples with evident injury to the lung tissue caused by the presence of silicon blocking, more subtle damage can be indistinguishable from that caused by LPS injection. Finally, the animals were ventilated for only two hours, and longer periods of MV that may be needed in clinical practice may lead to different outcomes.

## CONCLUSION

Our findings may suggest that atelectasis increases stress in the surrounding areas, favoring ventilator-induced lung injury in both previously healthy and injured lungs, despite protective ventilatory parameters. These results support the concept that reducing the number of interfaces between opened and closed alveolar units, which can be achieved by ventilatory strategies, such as positive end-expiratory pressure titration and prone positioning, might reduce ventilator-induced lung injury.
